# Left atrial ejection fraction and outcomes in heart failure with preserved ejection fraction

**DOI:** 10.1007/s10554-019-01684-9

**Published:** 2019-08-10

**Authors:** Prathap Kanagala, Jayanth R. Arnold, Adrian S. H. Cheng, Anvesha Singh, Jamal N. Khan, Gaurav S. Gulsin, Jing Yang, Lei Zhao, Pankaj Gupta, Iain B. Squire, Leong L. Ng, Gerry P. McCann

**Affiliations:** 1 Department of Cardiovascular Sciences, University of Leicester, National Institute for Health Research (NIHR) Leicester Biomedical Research Centre, Leicester, UK; 2 Aintree University Hospital, Liverpool, UK; 3 Kettering General Hospital NHS Foundation Trust, Kettering, UK; 4 Bristol-Myers Squibb, Princeton, NJ USA

**Keywords:** Cardiovascular magnetic resonance imaging, Heart failure with preserved ejection fraction, Prognosis, Left atrial ejection fraction, Biomarker

## Abstract

The aim of this study was to determine whether left atrial ejection fraction (LAEF) quantified with cardiovascular magnetic resonance (CMR) was different between heart failure with preserved ejection fraction (HFpEF) and controls, and its relation to prognosis. As part of our single-centre, prospective, observational study, 188 subjects (HFpEF n = 140, controls n = 48) underwent phenotyping with contrast-enhanced CMR, transthoracic echocardiography, blood sampling and six-minute walk testing. LAEF was calculated using the biplane method. Atrial fibrillation (AF) was present in 43 (31%) of HFpEF subjects. Overall, LAEF (%) was lower in HFpEF patients inclusive of AF (32 ± 16) or those in sinus rhythm alone (41 ± 12) compared to controls (51 ± 11), p < 0.0001. LAEF correlated inversely with maximal and minimal left atrial volumes indexed (*r* =  − 0.602, *r* =  − 0.762), and plasma N-terminal pro-atrial natriuretic peptide (*r* =  − 0.367); p < 0.0001. During median follow-up (1429 days), there were 67 composite events of all-cause death or hospitalization for heart failure (22 deaths, 45 HF hospitalizations) in HFpEF. Lower LAEF (below median) was associated with an increased risk of composite endpoints (Log-Rank: all p = 0.028; sinus p = 0.036). In multivariable Cox regression analysis, LAEF (adjusted hazard ratio [HR] 0.767, 95% confidence interval [CI] 0.591–0.996; p = 0.047) and indexed extracellular volume (HR 1.422, CI 1.015–1.992; p = 0.041) were the only parameters that remained significant when added to a base prognostic model comprising age, prior HF hospitalization, diastolic blood pressure, lung disease, NYHA, six-minute-walk-test-distance, haemoglobin, creatinine and B-type natriuretic peptide. CMR-derived LAEF is lower in HFpEF compared to healthy controls and is a strong prognostic biomarker.

**Electronic supplementary material:**

The online version of this article (10.1007/s10554-019-01684-9) contains supplementary material, which is available to authorized users.

## Introduction

Left atrial (LA) remodeling and dysfunction have been implicated in the pathophysiology of heart failure (HF) and are associated with poorer outcomes across a range of pathologies [1]. To date, the evidence base for such observations has largely been derived from echocardiography which is reliant upon adequate LA endocardial border definition for both volumetric and strain assessments [2]. Cardiovascular magnetic resonance imaging (CMR) affords superior spatial resolution, excellent contrast between blood pool and myocardium, has excellent reproducibility and is the current gold standard for LA volumetric [3] and functional assessment in sinus rhythm [4] or atrial fibrillation (AF) [5].

Heart failure with preserved ejection fraction (HFpEF) currently accounts for a significant proportion of all HF patients. Unlike heart failure with reduced ejection fraction (HFrEF), HFpEF still remains poorly understood and lacks proven, effective therapies [6]. Currently, prospective CMR studies assessing LA dysfunction in HFpEF are lacking. In this prospective, observational study of a well-characterized cohort with HFpEF, we assessed whether CMR-derived left atrial ejection fraction (LAEF) is different compared to controls and is of prognostic value.

## Materials and methods

### Study population

One hundred and fifty five patients with HFpEF were recruited in an observational, cohort study conducted at a single tertiary cardiac centre. HFpEF inclusion criteria were clinical or radiographic evidence of HF, left ventricular ejection fraction (LVEF) > 50% on transthoracic echocardiography (TTE) and age ≥ 18 years. The exclusion criteria were as previously described [7].

For comparison with HFpEF, 48 asymptomatic controls (age- and sex-matched) were recruited. Hypertensive subjects were included in this group (n = 22) since hypertension is highly prevalent in this age group of patients. The study was approved by the National Research Ethics Service (reference: 12/EM/0222). Written informed consent was obtained from all subjects prior to participation.

All subjects underwent comprehensive clinical assessment and blood sampling, TTE and CMR during the same visit. A standardized six minute walk test (6MWT) was used to quantify exercise capacity [8].

### Blood sampling

Blood was sampled for B-type natriuretic peptide ([BNP]-immunoassay, Siemens, Erlangen, Germany), haematocrit, haemoglobin and renal function (urea, creatinine). Plasma samples were analysed in a single batch for N-terminal pro-atrial natriuretic peptide (NT-proANP), a marker of atrial stress/stretch, using a Luminex® bead-based multiplex assay as previously described [9].

### Transthoracic echocardiography

Echocardiography was performed by accredited sonographers in accordance with American Society of Echocardiography guidelines using an iE33 system (Philips Medical Systems, Best, The Netherlands) as previously reported [7, 10]. LVEF was calculated using the biplane method or estimated visually where endocardial border definition was sub-optimal. Septal and lateral mitral annular diastolic velocities were averaged and used to derive E/E′ as an overall measure of diastolic function.

### CMR protocol

All CMR scans were performed on a 3-Tesla scanner (Siemens Skyra, Erlangen, Germany) with an 18-channel cardiac coil as previously detailed [7, 10]. Retrospective ECG gating was nominally used for image acquisition. In cases of arrhythmia, prospective gating was employed. In brief, the protocol comprised: standard long- and short-axis cine imaging; basal, mid-ventricular and apical short-axis T1 mapping pre- and post-contrast and late gadolinium enhancement (LGE) imaging at least 10 min after the final injection of contrast (Gadovist, Bayer Healthcare, Germany-total dose 0.15 mmol/kg). All cine images were acquired using balanced steady state free precession (SSFP) sequences and the following parameters: slice thickness 8 mm; distance factor 25%; image matrix 256 × 204 and segments amended according to heart rate ( < 70 b.p.m = 15 segments; 70–80 b.p.m = 12 segments; 80–100 b.p.m = 11 segments).

### CMR analysis

All images were analysed by a single operator (PK) blinded to clinical data, using *CVI42* software (Circle Cardiovascular Imaging, Calgary, Canada). Left ventricular volumes, EF and mass were calculated from the short-axis cine stack excluding papillary muscles and trabeculations [7]. Qualitative assessment of LGE images was undertaken by consensus of 2 experienced observers (PK, ASHC) and in cases of disagreement a third (GPM) for identifying the presence and pattern of focal fibrosis i.e. MI or non-MI fibrosis. Measures of diffuse myocardial fibrosis, namely ECV (extracellular volume) and iECV (ECV indexed to body surface area) were also calculated from mid-ventricular T1 mapping, as described by our group with excellent reproducibility [10]. Segments with MI or artefact were excluded from final T1 and ECV calculation, and segmental values were then averaged. Regions of focal non-MI fibrosis were included in our ECV (and iECV) calculations.

**Fig. 1:**
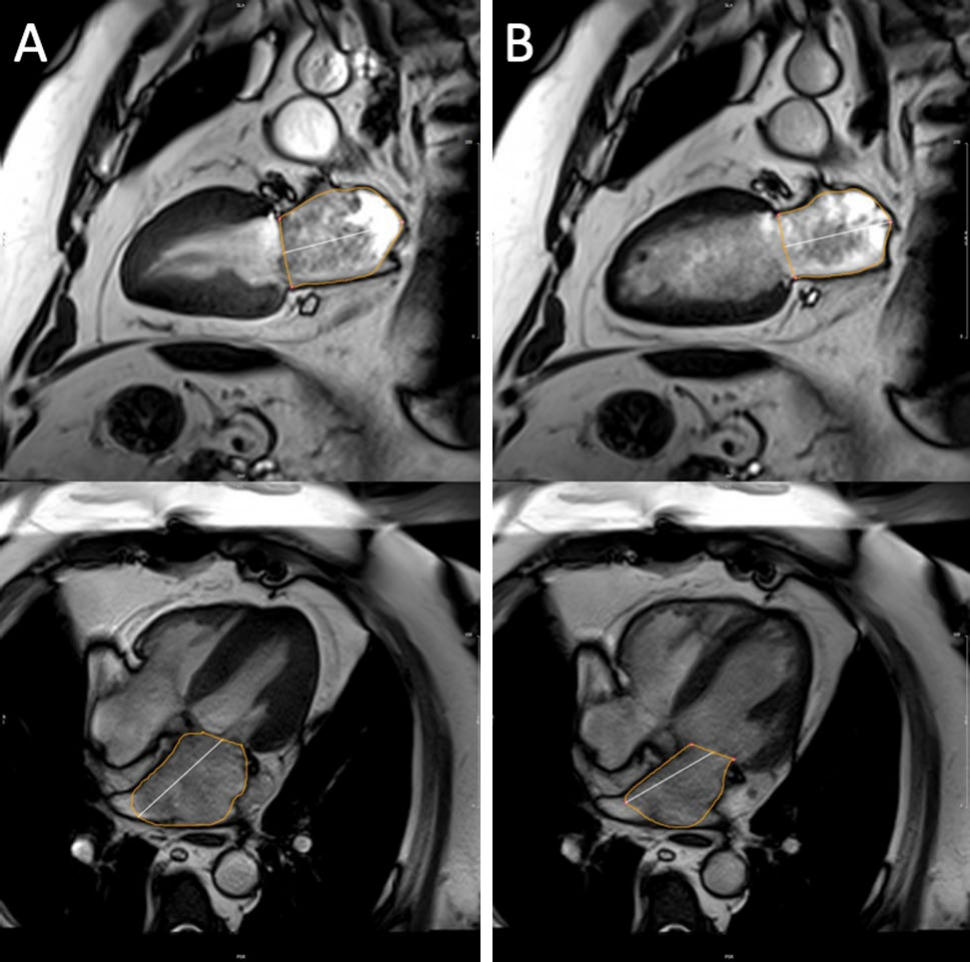
Calculation of left atrial ejection fraction. Cine 2- and 4-chamber images illustrating contoured maximum (**a**) and minimum (**b**) left atrial areas for volume (and ejection fraction) derivation

The biplane area-length method (excluding the appendage and pulmonary veins—Fig. 1) was employed for LA volumetric [11] and functional analysis [12]. The LA endocardial border was manually contoured in both the 2- and 4-chamber views with the mitral annulus serving as the division between the LA and LV. The maximum LA area was contoured in the frame immediately prior to mitral valve opening. The minimum LA area was contoured in the frame immediately after mitral valve closure. LA volumes (LAV) were calculated using the area-length method, where: volume = (0.85 × area^2^)/length. LAEF was derived as follows: LAEF = (LAVmax − LAVmin) /LAVmax. Surrogates of LA reservoir function i.e. reservoir volume ([LAVmax − LAVmin]) and LA conduit function i.e. conduit volume ([LV stroke volume − LA reservoir volume]) were also calculated. LAVImax > 40 ml/m^2^ was used to define LA dilation [11]. All volumetric and mass data were indexed to body surface area.

### Follow-up and endpoints

The primary endpoint was the composite of all-cause mortality or first HF hospitalization. Hospital databases and patient records were sourced to obtain outcome data. Patient follow-up was for a minimum of 12 months post-study entry. Only the first event was included in the outcome analysis.

### Statistical analysis

Statistical tests were performed using SPSS v22. Normality for continuous data was assessed using histograms, Q–Q plots and the Shapiro–Wilk test. Summary data are presented as mean (± standard deviation) or median (25–75% interquartile range). Between group differences were compared using the unpaired t-test, Mann–Whitney U test and the Chi-square test as appropriate. BNP and creatinine were log_10_ transformed before analysis. Pearson’s and Spearman’s correlations were performed to check for potential associations of LAEF with other continuous variables. Assessments of intra-observer and inter-observer variability for LA function were undertaken on ten randomly selected patients, a minimum of 4 weeks apart (by PK and JRA). Receiver operator characteristics (ROC) analyses were undertaken to assess the strength of the discriminatory capabilities of LAEF in distinguishing HFpEF from controls.

Kaplan–Meier analysis was undertaken to calculate event rates. Differences in survival curves were tested using the Log-Rank test. Cox proportional hazards analysis was undertaken to identify baseline variables associated with the composite endpoint. A base multivariable prognostic clinical model was created comprising clinical parameters shown to have historically strong prognostic importance in HF and univariable associations with the endpoint of p < 0.10. All remaining covariates associated with the endpoint at p < 0.10 were then entered into subsequent multivariable analysis to identify independent predictors using stepwise elimination methods. Continuous variables were Z-standardized to enable comparison of hazard ratios (HR) based upon one standard deviation increase in the predictor variable. The incremental prognostic benefit of LAEF beyond the base model was also assessed by comparing the area under the curves (AUCs) from ROC analysis.

## Results

### HFpEF versus controls

**Fig. 2:**
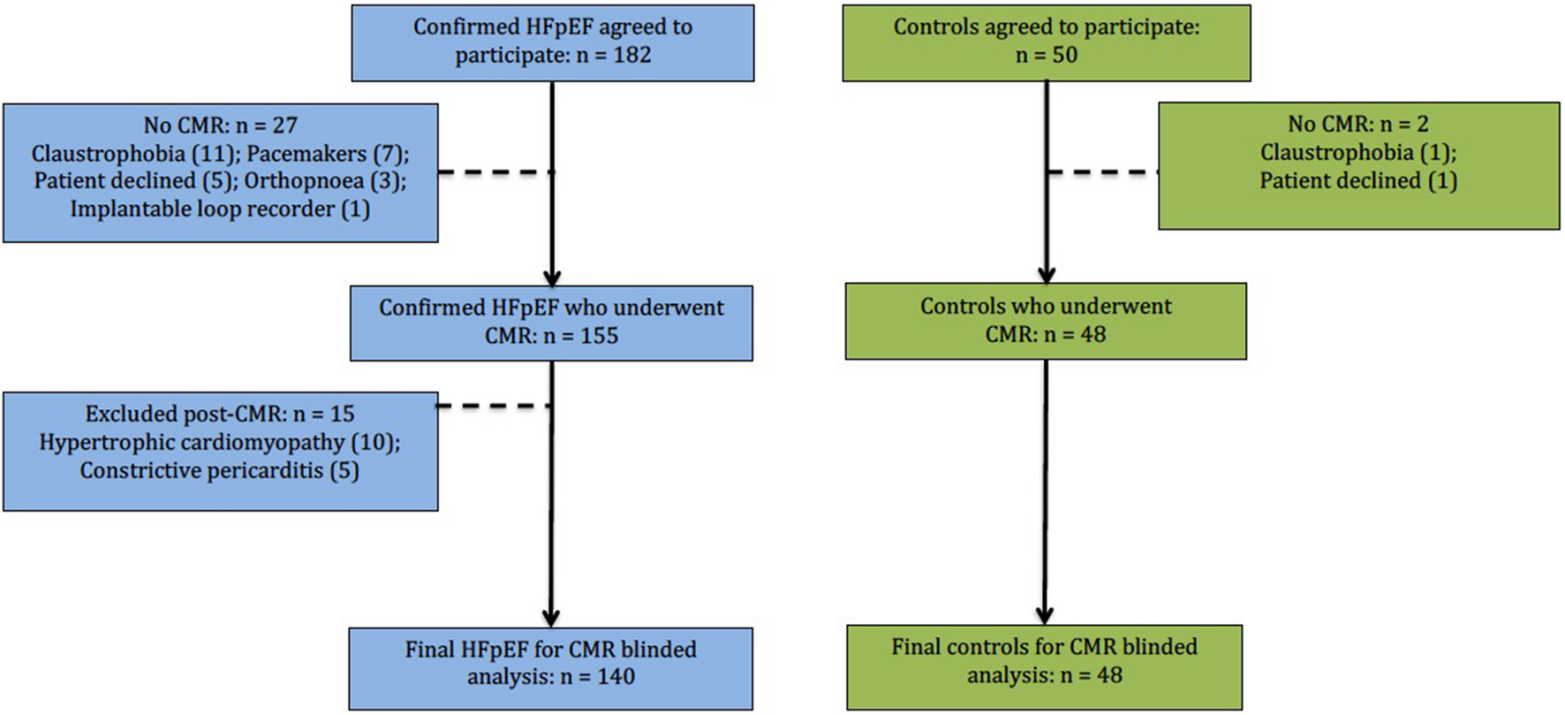
Study recruitment overview. Flow chart illustrating recruitment and CMR assessments. *CMR* cardiovascular magnetic resonance imaging, *HFpEF* heart failure with preserved ejection fraction

Following CMR, 15 HFpEF patients were diagnosed [7] with either hypertrophic cardiomyopathy (n = 10) or constrictive pericarditis (n = 5) and excluded from further analysis. Our final cohort thus comprised a total of 188 participants (Fig. 2). Baseline demographics and imaging characteristics are summarized in Tables 1 and 2.

**Table 1:** Baseline clinical characteristics

	HFpEF n = 140	Controls n = 48	p value
Demographics			
Age (years)	73 ± 9	73 ± 5	0.820
Male (%)	68 (49)	24 (50)	0.977
Clinical			
Heart rate (beats/min)	70 ± 14	68 ± 10	0.308
Systolic blood pressure (mmHg)	145 ± 25	151 ± 24	0.001
Diastolic blood pressure (mmHg)	74 ± 12	79 ± 10	0.006
Body mass index (kg/m^2^)	34 ± 7	25 ± 3	< 0.0001
Atrial fibrillation (%)	43 (31)	0 (0)	< 0.0001
Prior HF hospitalization (%)	92 (66)	NA	–
Diabetes (%)	75 (54)	0 (0)	< 0.0001
Hypertension (%)	127 (91)	22 (46)	< 0.0001
Angina (%)	23 (16)	0 (0)	0.003
Known myocardial infarction (%)	16 (11)	0 (0)	< 0.0001
Asthma or COPD (%)	24 (17)	3 (6)	0.134
Functional status			
NYHA III/IV (%)	43 (31)	NA	0.551
6MWT distance (m)	180 (120–250)	380 (350–440)	< 0.0001
Laboratory indices			
Urea (mmol/L)	9 ± 4	6 ± 1	< 0.0001
Creatinine (mmol/L)	89 (73–115)	71 (56–85)	< 0.0001
Haemoglobin (g/L)	129 ± 22	140 ± 15	0.003
BNP (ng/L)	136 (66–254)	33 (24–44)	< 0.0001
NTpro-ANP (pg/ml)	6443 (4362–8511)	4019 (3362–4475)	< 0.0001

Values are mean ± SD, n (%) or median, interquartile range. The p values are for the t-test or chi-square test

*BNP* B-type natriuretic peptide, *COPD* chronic obstructive pulmonary disease, *HF* heart failure, *HFpEF* heart failure with preserved ejection fraction, *IQR* interquartile range, *NA* not applicable, *NTpro-ANP* N-terminal pro-atrial natriuretic peptide, *NYHA* New York Heart Association class, *6MWT* six minute walk test

**Table 2:** Baseline imaging characteristics

	HFpEF n = 140	Controls n = 48	p value
Previous chest radiography			
Pulmonary oedema (%)	97 (69)	NA	–
Raised cardiothoracic ratio (%)	101 (72)	NA	–
Pleural effusion (%)	49 (35)	NA	–
Echocardiography			
E/E′	13 ± 6	9 ± 3	< 0.0001
CMR LV parameters			
LVEF (%)	56 ± 5	58 ± 5	0.019
LVEDVI (ml/m^2^)	79 ± 18	81 ± 14	0.409
LVESVI (ml/m^2^)	35 ± 10	34 ± 8	0.541
LV mass indexed (g/m^2^)	52 ± 15	46 ± 9	< 0.0001
LV mass/LVEDV	0.68 ± 0.16	0.57 ± 0.09	< 0.0001
Presence of MI (%)	23 (16)	0 (0)	< 0.0001
MI size (% of LV mass)	3.0 (1.3–4.6)	0 (0)	< 0.0001
Presence of non-MI focal fibrosis (%)	49 (35)	5 (10)	< 0.0001
Non-MI fibrosis size (% of LV mass)	2.9 (1.4–6.5)	2.4 (0.6–3.6)	0.002
Native myocardial T1 (ms)	1234 ± 73	1197 ± 91	0.021
Post-contrast myocardial T1 (ms)	461 ± 63	495 ± 85	0.011
ECV (%)	28 ± 4.6	25 ± 3.2	< 0.0001
iECV (ml/m^2^)	13.7 ± 4	10.9 ± 2.8	< 0.0001
CMR LA parameters			
Overall: all subjects including atrial fibrillation			
LAEF (%)	32 ± 16	51 ± 11	< 0.0001
Normal-sized LA (%)	50 (36)	33 (69)	< 0.0001
LAVImax (ml/m^2^)	53 ± 25	35 ± 12	< 0.0001
LAVImin (ml/m^2^)	38 ± 26	17 ± 8	< 0.0001
LA reservoir volume indexed ( ml/m^2^)	15 ± 7	17 ± 6	0.025
LA conduit volume indexed (ml/m^2^)	29 ± 9	30 ± 9	< 0.677

*ECV* extracellular volume, *iECV* indexed to body surface area, *extracellular volume LA* left atrium, *LAEF* left atrial ejection fraction, *LAVImax* left atrial maximal volume indexed to body surface area, *LAVImin* left atrial minimal volume indexed to body surface area, *LV* left ventricle, *LVEDVI* left ventricular end-diastolic volume indexed to body surface area, *LVEF* left ventricular ejection fraction, *LVESVI* left ventricular end-systolic volume indexed to body surface area, *MI* myocardial infarction

HFpEF and healthy controls were well matched for age (73 years) and sex. Approximately two-thirds of HFpEF patients had experienced prior hospital admissions for decompensated HF or had radiographic evidence of pulmonary congestion. Consistent with prior studies, HFpEF was frequently associated with co-morbidities including obesity, diabetes, hypertension and atrial fibrillation (AF). HFpEF patients had worse renal function and lower haemoglobin. A significant minority of HFpEF also had known ischaemic heart disease (22%, MI noted in 16%) and lung disease (17%). Furthermore, HFpEF patients had dramatically poorer exercise capacity (shorter 6MWT distance) and nearly one-third were in New York Heart Association (NYHA) III/IV.

### Imaging data

Indices of diastolic dysfunction as per European Society of Cardiology (ESC) guidelines i.e. BNP, E/E′, LAVImax, LV mass were significantly higher in HFpEF (Tables 1, 2). Compared to controls, the HFpEF group had greater concentric remodeling with increased LV mass/volume and a higher burden of both focal (MI and non-MI) and diffuse fibrosis (ECV, iECV); p < 0.0001 for all.

#### LA parameters

HFpEF subjects had higher plasma NT-proANP levels, larger atria and lower LAEF (32 ± 16) compared to controls: overall (51 ± 11), with hypertension (49 ± 12) and without hypertension (52 ± 11); p < 0.0001 for all (also see Table 2). There was no significant difference in LAEF between hypertensive and non-hypertensive controls (p = 0.324). Within HFpEF (Supplementary Table 1), AF was present in 31% and was associated with significantly higher LA volumes and lower LAEF (LAVImax 76 mls, LAVImin 66mls, LAEF 14%) compared to sinus rhythm (LAVImax 43mls, LAVImin 26mls, LAEF 41%, p < 0.0001). In HFpEF, normal LA size was noted in: 36% overall, 5% in AF and 50% in sinus rhythm. LAEF was lower in the presence of dilated LA compared to non-dilated LA: overall 26 ± 14 versus 44 ± 13; AF 13 ±  versus 36 ± 17; sinus rhythm 37 ± 9 versus 45 ± 13, p < 0.0001 for all.

In the whole cohort, a LAEF threshold value below 44% best discriminated HFpEF from controls using maximal sensitivity–specificity analysis; ROC-AUC 0.794, p < 0.00001. In sub-group analysis of sinus rhythm subjects, the same LAEF threshold yielded a ROC-AUC of 0.727, p < 0.00001.

#### LAEF correlations

**Fig. 3:**
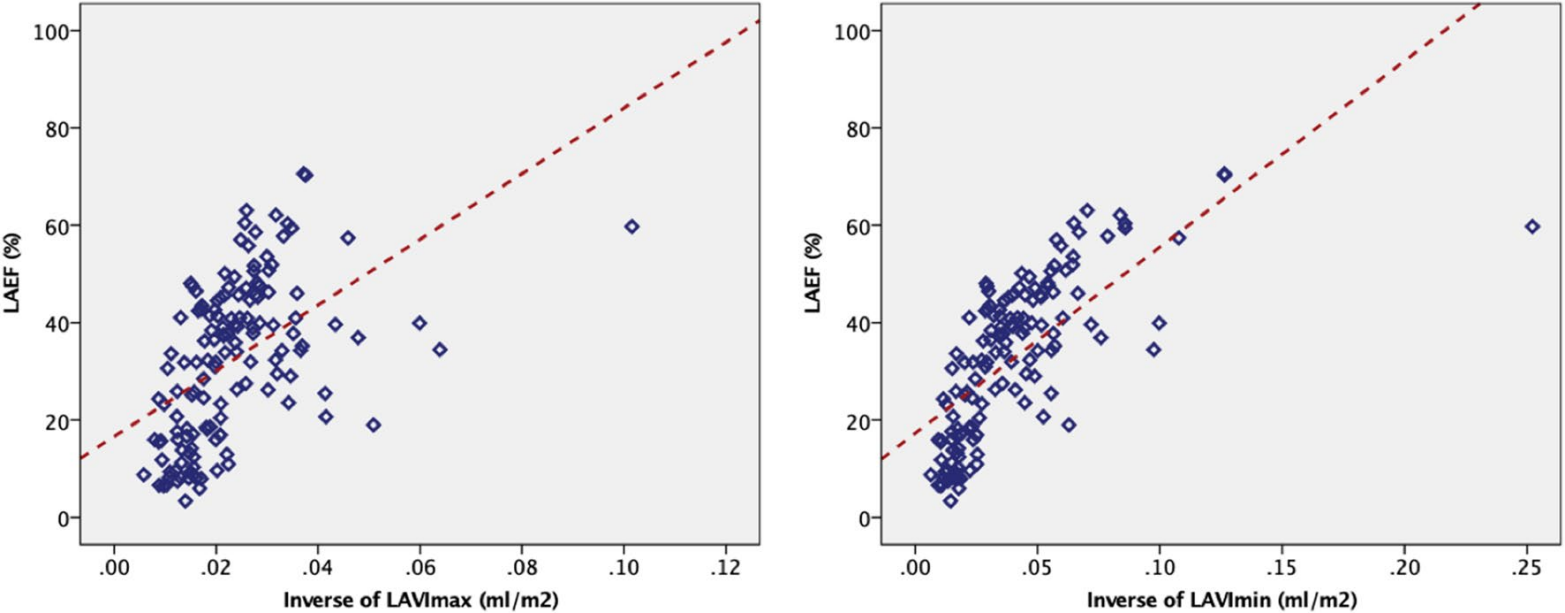
Associations of left atrial ejection fraction with left atrial volumes. Scatter plot illustrating the relationship between left atrial ejection fraction (LAEF) and the inverse of: maximum left atrium volume indexed-LAVImax (left panel) or minimum left atrium volume indexed-LAVImin (right panel)

LAEF correlated inversely with echocardiographic E/E′ (Pearson’s *r* =  − 0.247, p = 0.001). There were further moderate to strong inverse correlations between LAEF, NTproANP (Spearman’s *r* =  − 0.367) and LA volumes (LAVImax Pearson’s *r* =  − 0.602, LAVImin *r* =  − 0.762, see Fig. 3); p < 0.0001 for all. As LAEF diminished, LA volumes increased. Linear fit models of LAEF against the inverse of LAVImax (r^2^ = 0.253, p < 0.0001) and LAVImin (r^2^ = 0.485, p < 0.0001) are illustrated in Fig. 3. iECV did not correlate with LAEF (Pearson’s *r* =  − 0.067; p = 0.527).

#### Inter-observer and intra-observer assessments

Inter-observer and intra-observer variability agreements for LA volumes and LAEF were excellent (intra-class correlation coefficients 0.95–0.99; Supplementary Table 2).

### Survival analysis

During median follow-up of 1429 days (1157–1657), there were a total of 67 composite events (48%, 22 deaths, 45 HF hospitalizations) in patients with HFpEF. The event rate was higher in the AF sub-group than in sinus rhythm (55.8% vs 44.3%, p = 0.210). There were no events in the control group. No subjects were lost to follow-up.

#### Cox regression analysis

On univariable analysis comprising all HFpEF subjects, 18 parameters were associated with adverse outcomes (Table 3). AF was not significantly associated with outcomes on univariable analysis (p = 0.221). Plasma urea and creatinine exhibited collinearity. Of the urivariable predictors, nine variables (age, prior HF hospitalization, diastolic blood pressure, lung disease, NYHA class, 6MWT distance, haemoglobin, creatinine and BNP) were entered into a base clinical model. Of the remaining variables with univariate endpoint association of p < 0.10, LAEF (adjusted hazard ratio [HR] 0.767, 95% confidence interval [CI] 0.591–0.996; p = 0.047) and iECV (HR 1.422, CI 1.015–1.992; p = 0.041) were the only parameters to retain statistical significance on multivariable analysis in addition to the base model.

**Table 3:** Univariable predictors for the composite endpoint of death and/or hospitalization with heart failure

	Hazard ratio (95% CI)	P value
Univariable predictors of outcome		
Clinical		
Age^a^	1.386 (1.084–1.772)	0.009
Average DBP^a^	0.650 (0.492–0.858)	0.002
Prior HF hospitalization^a^	2.902 (1.553–5.423)	0.001
Lung disease^a^	1.891 (1.077–3.321)	0.027
NYHA III/IV^a^	1.703 (1.044–2.780)	0.033
6MWT distance^a^	0.659 (0.465–0.934)	0.019
Clinical blood samples		
Urea (mmol/L)	1.197 (0.971–1.475)	0.092
Log creatinine (mmol/L)^a^	1.312 (1.048–1.642)	0.018
Haemoglobin (g/L)^a^	0.727 (0.570–0.927)	0.010
Log BNP (ng/L)^a^	1.471 (1.081–2.000)	0.014
NTproANP	1.314 (1.029–1.677)	0.028
Imaging		
E/E′	1.459 (1.143–1.862)	0.002
LV mass index	1.296 (1.005–1.671)	0.046
LAVImax	1.237 (0.992–1.543)	0.059
LGE MI	1.670 (0.926–3.012)	0.088
ECV	1.519 (1.076–2.145)	0.018
iECV	1.516 (1.105–2.079)	0.010
LAEF	0.726 (0.568–0.927)	0.010

Abbreviations are as for Tables 1 and 2; Hazard ratios are based upon one standard deviation increase in the predictor variable for continuous variables which are Z-standardized

*CI* confidence interval

^a^Parameters entered into the base clinical multivariable model

#### Kaplan–Meier analysis

**Fig. 4:**
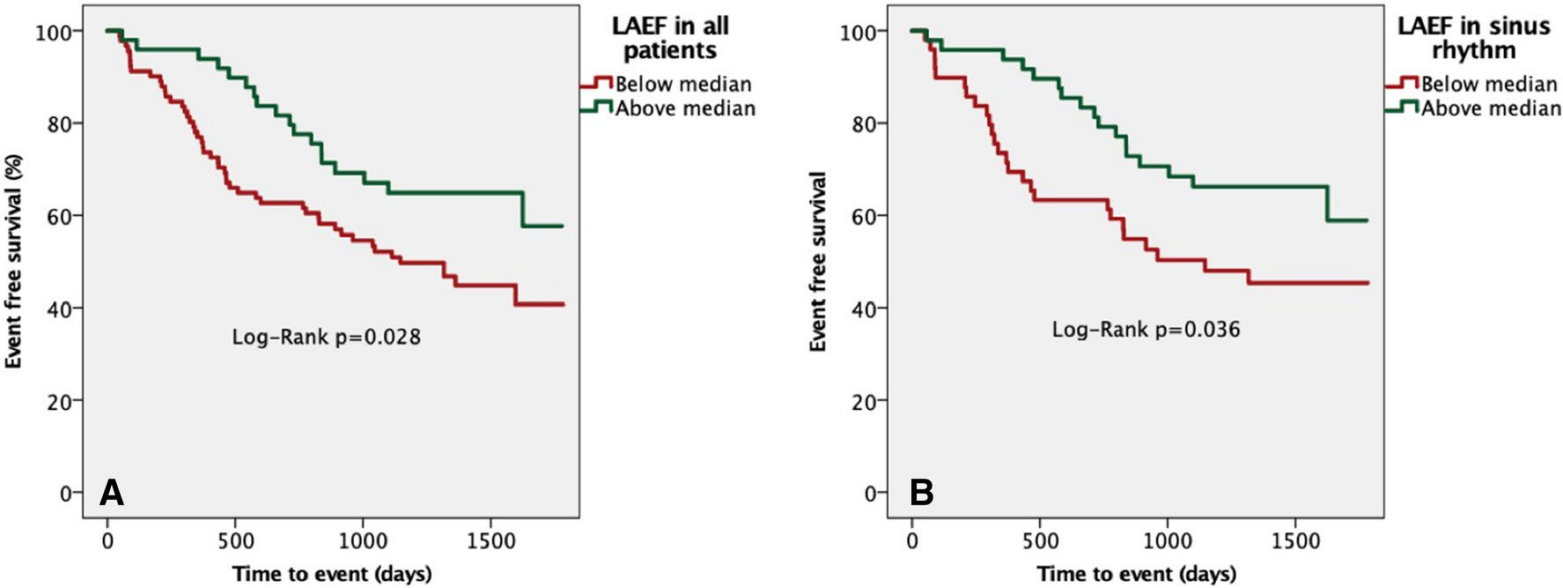
Survival analysis stratified according to median left atrial ejection fraction. Kaplan–Meier analysis stratified according to median left atrial ejection fraction for the composite endpoint of death and/or hospitalization with heart failure in **a** all subjects and **b** in sinus rhythm only

Kaplan–Meier survival plots according to LAEF for all patients or sinus rhythm alone are shown in Fig. 4. A lower LAEF (below median) was associated with increased risk of death or HF hospitalization (all Log-Rank p = 0.028; sinus rhythm Log-Rank p = 0.036). i.e. the text should match the part labels.

#### ROC analysis

The AUCs (see Supplementary Fig. 1) of the base model and LAEF for predicting outcomes were 0.790 (p < 0.0001) and 0.634 (p = 0.008) respectively. The combination of the base model and LAEF yielded a higher AUC of 0.806 (p < 0.0001). The AUC for iECV was 0.611 (p = 0.078).

## Discussion

This study prospectively evaluated the prognostic relevance of CMR-derived LAEF in a well-phenotyped cohort of HFpEF and healthy subjects. The principal findings are that (a) LAEF is lower in HFpEF compared to age- and sex-matched healthy controls (b) LAEF is associated with LA volumes and plasma biomarkers of atrial stress/stretch (c) inclusive of AF or sinus rhythm alone, lower LAEF is associated with adverse outcomes in HFpEF and (d) CMR-LAEF is also an independent prognostic marker in HFpEF.

## LAEF and HFpEF

Our work adds to a growing body of evidence implicating LA remodeling and dysfunction in HF [1]. Impaired LA function has previously been noted in conditions associated with HFpEF (e.g. diabetes, hypertension) even in the presence of normal LA size [13]. Furthermore, LAEF is reportedly lower in HFpEF compared to hypertensive subjects with LVH, corroborating our findings [14]. Diminished LA contractile reserve as a predictor of exercise incapacity has also been shown in subjects with preserved LV ejection fraction with [15] and without heart failure [16].

Current ESC guidelines advocate measurement of LA volumes and LV mass in all subjects with suspected HFpEF [6]. However, these measures are reliant on image quality and adequate endocardial border definition, unfortunately lacking in a third of HF cases when assessed with TTE [17]. Excellent spatial resolution and contrast, as well as the ability to scan in any image plane make CMR the current imaging gold standard [17]. Current imaging diagnostic criteria provide cut-offs for LAVImax and LV mass that are echocardiography-based and do not routinely incorporate CMR [6]. In our study, ROC analyses confirmed the strong signal from LAEF in discriminating HFpEF from controls. The reasons for this are likely multiple. Firstly, LAEF reduction might be a more precise reflection of elevated filling pressures than the other traditional surrogate imaging markers of chronic diastolic dysfunction [18]. Similar to our study, published literature has demonstrated normal-sized LA in approximately one-third of HFpEF subjects [19]. Our findings of reduced LAEF even in the presence of normal-sized atria reaffirms prior observations that LA dysfunction likely precedes overt LA remodeling in HFpEF [20]. Towards the other end of the spectrum, with worsening LA dilatation (and likely chronic LV&LA pressure overload), we have also demonstrated a close relationship between LA systolic function and volumes akin to the Frank-Starling mechanism i.e. LAEF reduces significantly more at higher volumes as contractile reserve becomes exhausted [12, 21]. In our subjects, more specific derangements in both reservoir (increased LAVmax and reservoir volume) and booster pump (increased LAVmin) function were also noted. LA reservoir function may be compromised by reduced LA compliance and LV longitudinal dysfunction typical of HFpEF [22]. In addition, LV diastolic dysfunction and concomitant elevated filling pressures further contribute to ineffective LA active emptying through increasing LA afterload and wall tension [23]. Compensatory improvements in conduit function may in part explain the lack of difference in conduit volume between HFpEF and controls in our study [24].

## LAEF as a potential prognostic biomarker

Our prospective study shows CMR-derived LAEF is an independent prognostic marker in HFpEF, both with and without AF. Previously, TTE-based observational studies [25] and HFpEF clinical trials [20, 23] have highlighted perturbed LA function as a marker of adverse outcomes. Using indices of LA strain measured by speckle tracking, LA dysfunction was independently associated with either prior [20] or subsequent [23] HF hospitalizations and death [25]. In a further retrospective TTE study involving both HFpEF and HFrEF, LAEF was independently associated with death only in HFpEF [21]. However, in the latter study, the groups were not evenly matched and controls comprised subjects referred for cardiac catheterization and were perhaps not truly representative of a healthy comparator group. In the one published CMR study to date evaluating the role of LA function in HF (heterogeneous population primarily comprising HFrEF), LAEF independently predicted mortality and incident AF. However, this retrospective study was again limited by referral bias, lacking a control group and excluding subjects who were in AF (nearly one-third) [12].

The potential value of LAEF as a prognostic biomarker may not be confined to HF alone. In a previous study of 312 subjects free of HF, who were in sinus rhythm and of similar age to our cohort, LAEF and LA strain were independent predictors of outcomes including future development of AF, HF and cardiovascular death [26]. All of the aforementioned studies however share intrinsic limitations of TTE [4].

Beyond HF, CMR data also further support LA dysfunction as a marker of outcomes. Similar to our findings, the incremental prognostic value of LA function beyond LAVImax has previously been shown in a prospective study of asymptomatic subjects from the general population [27] and in chronic hypertensive subjects without prevalent cardiovascular disease [28]. These findings suggest that LAEF also reflects a more advanced state of LA remodeling than LA dilation alone [1]. In another population study, LA strain using CMR feature tracking was independently associated with future development of incident heart failure [29].

## LAEF and AF

The association between LA dilation and AF and their attendant cardiovascular risk is well recognized [1]. In HF, AF risk is also known to increase with diminishing LAEF [12]. Interestingly, in our study, AF was not associated with adverse outcomes even though event rates were higher in this sub-group and LAEF was significantly lower compared to those in sinus rhythm. This suggests that LAEF exerts its influence on outcomes through alternate mechanisms, either directly or indirectly [24, 27]. LA dysfunction as a mediator of pulmonary vascular damage, RV dysfunction and progressive biventricular failure has also been proposed [21]. Additional reports have also highlighted that LA dysfunction in the presence of AF has incremental thromboembolic and mortality risk, beyond the CHADS2 (congestive heart failure = 1, hypertension = 1, age ≥ 75 = 1, stroke/transient ischaemic attack = 2) score. Furthermore, LA dysfunction (using echo strain measures) predicts the success of restoring and maintaining sinus rhythm following either direct-current cardioversion or AF ablation [1].

## Potential implications of our study

Our study reaffirms the pathophysiological role of LA dysfunction in HFpEF. CMR-measured biplane LAEF is simple, reproducible and provides prognostic information which are strengths for consideration as a potential biomarker. CMR is becoming increasingly accessible and may more reliably discriminate breathless individuals with equivocal BNP levels and suboptimal echocardiographic imaging windows (especially HFpEF) [6, 17]. Recent data have also suggested that LA dysfunction may be a potential therapeutic target [24]. While our study suggests that iECV and LA function are not related, ongoing clinical trial data may shed further insight into whether myocardial fibrosis regression in HFpEF (with the anti-fibrotic agent Pirfenidone) may alter LA function as a secondary outcome measure [30].

## Limitations

This is a single centre study and the results should be confirmed in additional HFpEF cohorts. We used a pragmatic approach to define our HFpEF population to reflect a real world setting as opposed to latest ESC guidelines [6]. The presence of diastolic dysfunction was not a pre-requisite for study inclusion since recent contemporary clinical trial data have highlighted normal diastolic function in approximately a third of such patients [31]. Our data does however also provide compelling evidence (natriuretic peptides) that our patient cohort truly had HF and the event rates (48%) are similar to that of previous outcome studies in HFpEF. Our control group included hypertensive subjects and was therefore not totally free of cardiovascular disease. However, LAEF was also lower in HFpEF compared to both hypertensive and non-hypertensive controls.

## Conclusions

CMR-derived LAEF is lower in HFpEF compared to age- and sex-matched controls and independently predicts outcomes.

## Electronic supplementary material

Below is the link to the electronic supplementary material.

Supplementary file1 (PDF 162 kb)

Supplementary file2 (PDF 175 kb)
