# Supplementary material for: Left atrial ejection fraction and outcomes in heart failure with preserved ejection fraction
Source: Int J Cardiovasc Imaging. 2019 Aug 10;36(1):101–10. doi: 10.1007/s10554-019-01684-9 (PMC6942575; doi:10.1007/s10554-019-01684-9)
Supplement: Supplementary file 1 — Supplementary file1 (PDF 162 kb) [file 10554_2019_1684_MOESM1_ESM.pdf]

Title

Left atrial ejection fraction and outcomes in heart failure with preserved ejection fraction

Journal

The International Journal of Cardiovascular Imaging

Names of authors and affiliations

Prathap Kanagala[a, b] – MBBS, PhD pkk12@leicester.ac.uk

Jayanth R. Arnold[a] – BMBChB, DPhil jra14@leicester.ac.uk

Adrian S.H. Cheng[c] – MBBS, MD adrianshcheng@gmail.com

Anvesha Singh[a] – MBChB, PhD as707@leicester.ac.uk

Jamal N. Khan[a] – MBChB, PhD mally777@hotmail.com

Gaurav S. Gulsin[a] – gg149@leicester.ac.uk

Jing Yang[d] – PhD jing.yang1@bms.com

Lei Zhao[d]– PhD lei.zhao2@bms.com

Pankaj Gupta[a] – DPB, FRCPath pankaj_gupta54@hotmail.com

Iain B. Squire[a] – MBChB, MD is11@leicester.ac.uk

Leong L. Ng[a] – MB B Chir, MD lln1@leicester.ac.uk

Gerry P. McCann[a] – MBChB, MD gpm12@leicester.ac.uk

From the Department of Cardiovascular Sciences, University of Leicester, National Institute for Health Research (NIHR) Leicester Biomedical Research Centre, Leicester, United Kingdom[a], Aintree University Hospital, Liverpool, United Kingdom[b] and Kettering General

Hospital NHS Foundation Trust, Kettering, United Kingdom[c]. Bristol-Myers Squibb, Princeton, New Jersey, USA[d]

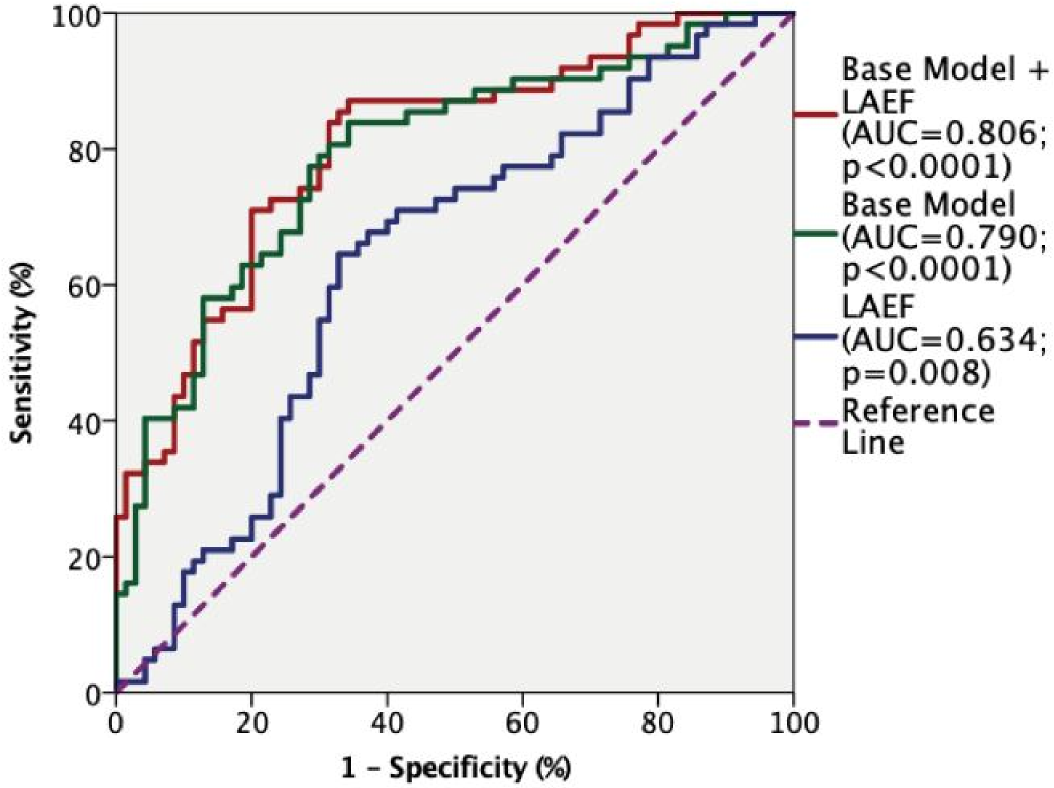

Online Resource Supplementary Fig 1. Receiver operator characteristics analysis of the base clinical model, LAEF and the combined model to predict outcomes
