# Supplementary material for: Left atrial ejection fraction and outcomes in heart failure with preserved ejection fraction
Source: Int J Cardiovasc Imaging. 2019 Aug 10;36(1):101–10. doi: 10.1007/s10554-019-01684-9 (PMC6942575; doi:10.1007/s10554-019-01684-9)
Supplement: Supplementary file 2 — Supplementary file2 (PDF 175 kb) [file 10554_2019_1684_MOESM2_ESM.pdf]

Title

Left atrial ejection fraction and outcomes in heart failure with preserved ejection fraction

Journal

The International Journal of Cardiovascular Imaging

Names of authors and affiliations

Prathap Kanagala[a, b] – MBBS, PhD pkk12@leicester.ac.uk

Jayanth R. Arnold[a] – BMBChB, DPhil jra14@leicester.ac.uk

Adrian S.H. Cheng[c] – MBBS, MD adrianshcheng@gmail.com

Anvesha Singh[a] – MBChB, PhD as707@leicester.ac.uk

Jamal N. Khan[a] – MBChB, PhD mally777@hotmail.com

Gaurav S. Gulsin[a] – gg149@leicester.ac.uk

Jing Yang[d] – PhD jing.yang1@bms.com

Lei Zhao[d]– PhD lei.zhao2@bms.com

Pankaj Gupta[a] – DPB, FRCPath pankaj_gupta54@hotmail.com

Iain B. Squire[a] – MBChB, MD is11@leicester.ac.uk

Leong L. Ng[a] – MB B Chir, MD lln1@leicester.ac.uk

Gerry P. McCann[a] – MBChB, MD gpm12@leicester.ac.uk

From the Department of Cardiovascular Sciences, University of Leicester, National Institute for Health Research (NIHR) Leicester Biomedical Research Centre, Leicester, United Kingdom[a], Aintree University Hospital, Liverpool, United Kingdom[b] and Kettering General

Hospital NHS Foundation Trust, Kettering, United Kingdom[c]. Bristol-Myers Squibb, Princeton, New Jersey, USA[d]

Online Resource Supplementary Materials

Online Resource Supplementary Table 1. CMR LA parameters according to atrial fibrillation and sinus rhythm

	HFpEF<br>n = 140	Controls<br>n = 48	p value
Atrial fibrillation subjects only			
LAEF (%)	14±7	NA	-
Normal-sized LA (%)	2 (5)	NA	-
LAVImax (ml/m^2)	76±27	NA	-
LAVImin (ml/m^2)	66±25	NA	-
LA reservoir volume indexed (ml/m^2)	10±5	NA	-
LA conduit volume indexed (ml/m^2)	32±10	NA	-
Sinus rhythm subjects only			
LAEF (%)	41±12	51±11	<0.0001
Normal-sized LA (%)	48 (50)	33 (69)	0.037
LAVImax (ml/m^2)	43±17	35±12	<0.001
LAVImin (ml/m^2)	26±13	17±8	<0.0001
LA reservoir volume indexed (ml/m^2)	17±6	17±6	0.791
LA conduit volume indexed (ml/m^2)	28±8	30±9	0.136
Abbreviations are as for main Table 2			

Online Resource Supplementary Table 2. Intra-observer and inter-observer assessments for left atrial volumes and left atrial ejection fraction

Parameter	Observer 1 Mean ± SD	Observer 2 Mean ± SD	Mean difference ± SD	ICC	Variability (1 – ICC)	Co-efficient of variation	95% Limits of Agreement
Intra-observer							
LAV min (ml)	70 ± 45	71 ± 44	1 ± 4	0.99	0.01	5.4	-7 to 8
LAV max (ml)	99 ± 48	101 ± 49	2 ± 5	0.99	0.01	4.8	-7 to 12
LAEF (%)	33 ± 13	33 ± 13	0.1 ± 3	0.98	0.02	9.4	-6 to 6
Inter-observer							
LA min (ml)	70 ± 45	71 ± 46	0.7 ± 5	0.99	0.01	6.8	-9 to 10
LA max (ml)	99 ± 48	102 ± 47	3 ± 6	0.99	0.01	6.3	-10 to 15
LAEF (%)	33 ± 13	35 ± 16	2 ± 4	0.95	0.05	12.2	-6 to 10
Abbreviations are as for main Table 2 and: ICC = intra-class correlation coefficient							
